# Supplementary material for: The Impact of COVID-19 Pandemic on the Clinical Practice Patterns in Atrial Fibrillation: A Multicenter Clinician Survey in China
Source: J Clin Med. 2022 Oct 31;11(21):6469. doi: 10.3390/jcm11216469 (PMC9653826; doi:10.3390/jcm11216469)
Supplement: Supplementary file 1 [file jcm-11-06469-s001.zip › jcm-1908443-supplementary/Supplement S1.pdf]

Dear colleague,

Welcome to the survey entitled “Clinician Perspectives on the Impact of COVID-19 Pandemic on the Clinical Practice Patterns in Atrial Fibrillation”. The purpose of this survey is to help us better understand atrial fibrillation (AF) treatment practices during the COVID-19 pandemic. Especially, the clinical decisions for emergencies such as hemodynamic instability induced by AF were not involved in this clinical survey. Some rarely used AF treatments, such as AF surgery, hybrid surgical/catheter ablation procedures, atrioventricular node ablation and pacing, and surgical left atrial appendage exclusion were also not included in the questionnaire. In this questionnaire, “previous non-pandemic period” refers to the year of 2019 before COVID-19 pandemic outbreak. The data collected in this survey will be kept anonymously. This survey includes demographic questions and hypothetical clinical questions. It should take you approximately 20 minutes to complete.

1. In which province is your medical institution located?

2. What is the classification of the hospital you work in? [single choice]

A. Primary general hospital

B. Secondary general hospital

C. Tertiary general hospital

D. Cardiovascular hospital

3. How many years have you been practicingcardiology? [single choice]

A. ≤5

B. 6–10

C. 11–20

D. >20

4. What is your subspecialty in cardiology? [multiple choice]

A.Arrhythmias

B. Coronary heart disease

C. Congenital heart disease / structural heart disease

D. Heart failure

E. Hypertension

F. Dyslipidemia

G. Critical cardiovascular diseases

H. Cardiovascular diseases without detailed subspecialty

I. Other subspecialty______

5. What is your subspecialty in interventional therapy? [multiple choice]

A. Electrophysiology

B. Coronary artery intervention therapy

C. Cardiac device implantation

D. Interventional therapy for congenital heart disease

E. Interventional therapy for peripheral vascular diseases

F. Other interventional therapy

G. Not an interventional physician

6. What is the average daily number of newlyreported COVID-19 cases in your **province** in the past week? [single choice]

A. ≤10

B. 11–100

C. 101–500

D. 501–1000

E. >1000

7. What is the average daily number of newly reported COVID-19 cases in your **city**

in the past week? [single choice]

A. ≤10

B. 11–100

C. 101–500

D. 501–1000

E. >1000

8. Compared with the previous non-pandemic period, how did the COVID-19 pandemic affect the number of **outpatients** with AF in your hospital in the past month? [single choice]

A. No significant change

B. Increased by less than 20%

C. Increased by 20%–50%

D. Increased by 50%–100%

E. Increased more than100%

F. Decreased by less than 20%

G. Decreased by 20%–50%

H. Decreased by 50%–100%

I. Decreased more than100%

9. Compared with the previous non-pandemic period, how did the COVID-19 pandemic affect the number of **inpatients** with AF in your hospital in the past month? [single choice]

A. No significant change

B. Increased by less than 20%

C. Increased by 20%–50%

D. Increased by 50%–100%

E. Increased more than100%

F. Decreased by less than 20%

G. Decreased by 20%–50%

H. Decreased by 50%–100%

I. Decreased more than100%

10. Compared with the previous non-pandemic period, how did the COVID-19 pandemic affect the number of **patients with AF who underwent catheter ablation** in your hospital in the past month? [single choice]

A. No significant change

B. Increased by less than 20%

C. Increased by 20%–50%

D. Increased by 50%–100%

E. Increased more than100%

F. Decreased by less than 20%

G. Decreased by 20%–50%

H. Decreased by 50%–100%

I. Decreased more than100%

11. During the COVID-19 pandemic, what therapeutic strategy would you choose if the **paroxysmal** AF patient was **positive** for COVID-19? [multiple choice]

A. Management of cardiovascular risk factors (CRS) and concomitant diseases

B. Pharmacotherapy in stroke prevention (anticoagulant/antiplatelet according to stroke risk and bleeding risk assessment)

C.Heart rate control with medication

D. Rhythm control with medication

E. Electrical cardioversion

F. Catheter ablation for asymptomatic COVID-19 positive cases

G. Percutaneous left atrial appendage occlusion for asymptomatic COVID-19 positive cases

H.Other therapeutic advice______

12. During the COVID-19 pandemic, what therapeutic strategy would you choose if the **paroxysmal** AF patient was **negative** for COVID-19? [multiple choice]

A. Management of cardiovascular risk factors (CRS) and concomitant diseases

B. Pharmacotherapy in stroke prevention (anticoagulant/antiplatelet according to stroke risk and bleeding risk assessment)

C.Heart rate control with medication

D. Rhythm control with medication

E. Electrical cardioversion

F. Catheter ablation

G. Percutaneous left atrial appendage occlusion

H.Other therapeutic advice______

13. During the **previous non-pandemic period**, what therapeutic strategy would you choose for the **paroxysmal** AF patient? [multiple choice]

A. Management of cardiovascular risk factors (CRS) and concomitant diseases

B. Pharmacotherapy in stroke prevention (anticoagulant/antiplatelet according to stroke risk and bleeding risk assessment)

C.Heart rate control with medication

D. Rhythm control with medication

E. Electrical cardioversion

F. Catheter ablation

G. Percutaneous left atrial appendage occlusion

H.Other therapeutic advice______

14. During the COVID-19 pandemic, what therapeutic strategy would you choose if the **persistent** AF patient was **positive** for COVID-19? [multiple choice]

A. Management of cardiovascular risk factors (CRS) and concomitant diseases

B. Pharmacotherapy in stroke prevention (anticoagulant/antiplatelet according to stroke risk and bleeding risk assessment)

C.Heart rate control with medication

D. Rhythm control with medication

E. Electrical cardioversion

F. Catheter ablation for asymptomatic COVID-19 positive cases

G. Percutaneous left atrial appendage occlusion for asymptomatic COVID-19 positive cases

H.Other therapeutic advice______

15. During the COVID-19 pandemic, what therapeutic strategy would you choose if the **persistent** AF patient was **negative** for COVID-19? [multiple choice]

A. Management of cardiovascular risk factors (CRS) and concomitant diseases

B. Pharmacotherapy in stroke prevention (anticoagulant/antiplatelet according to stroke risk and bleeding risk assessment)

C.Heart rate control with medication

D. Rhythm control with medication

E. Electrical cardioversion

F. Catheter ablation

G. Percutaneous left atrial appendage occlusion

H.Other therapeutic advice______

16. During the **previous non-pandemic period**, what therapeutic strategy would you choose for the **persistent** AF patient? [multiple choice]

A. Management of cardiovascular risk factors (CRS) and concomitant diseases

B. Pharmacotherapy in stroke prevention (anticoagulant/antiplatelet according to stroke risk and bleeding risk assessment)

C.Heart rate control with medication

D. Rhythm control with medication

E. Electrical cardioversion

F. Catheter ablation

G. Percutaneous left atrial appendage occlusion

H.Other therapeutic advice______

17. During the COVID-19 pandemic, what therapeutic strategy would you choose if the **long-standing persistent or permanent** AF patient was **positive** for COVID-19? [multiple choice]

A. Management of cardiovascular risk factors (CRS) and concomitant diseases

B. Pharmacotherapy in stroke prevention (anticoagulant/antiplatelet according to stroke risk and bleeding risk assessment)

C.Heart rate control with medication

D. Rhythm control with medication

E. Electrical cardioversion

F. Catheter ablation for asymptomatic COVID-19 positive cases

G. Percutaneous left atrial appendage occlusion for asymptomatic COVID-19 positive cases

H.Other therapeutic advice______

18. During the COVID-19 pandemic, what therapeutic strategy would you choose if the **long-standing persistent or permanent** AF patient was **negative** for COVID-19? [multiple choice]

A. Management of cardiovascular risk factors (CRS) and concomitant diseases

B. Pharmacotherapy in stroke prevention (anticoagulant/antiplatelet according to stroke risk and bleeding risk assessment)

C.Heart rate control with medication

D. Rhythm control with medication

E. Electrical cardioversion

F. Catheter ablation

G. Percutaneous left atrial appendage occlusion

H.Other therapeutic advice______

19. During the **previous non-pandemic period**, what therapeutic strategy would you choose for the **long-standing persistent or permanent** AF patient? [multiple choice]

A. Management of cardiovascular risk factors (CRS) and concomitant diseases

B. Pharmacotherapy in stroke prevention (anticoagulant/antiplatelet according to stroke risk and bleeding risk assessment)

C.Heart rate control with medication

D. Rhythm control with medication

E. Electrical cardioversion

F. Catheter ablation

G. Percutaneous left atrial appendage occlusion

H.Other therapeutic advice______

20. Regarding the treatment strategies of **first diagnosed AF** patients, what level do you think the COVID-19 pandemic has impact on you? [single choice]

A. Almost no impact

B. Mild impact

C. Moderate impact

D. Serious impact

21. Regarding the treatment strategies of **paroxysmal AF** patients, what level do you think the COVID-19 pandemic has impact on you? [single choice]

A. Almost no impact

B. Mild impact

C. Moderate impact

D. Serious impact

22. Regarding the treatment strategies of **peresistent AF** patients, what level do you think the COVID-19 pandemic has impact on you? [single choice]

A. Almost no impact

B. Mild impact

C. Moderate impact

D. Serious impact

23. Regarding the treatment strategies of **long-standing persistent AF** patients, what level do you think the COVID-19 pandemic has impact on you? [single choice]

A. Almost no impact

B. Mild impact

C. Moderate impact

D. Serious impact

24. Regarding the treatment strategies of **permanent AF** patients, what level do you think the COVID-19 pandemic has impact on you? [single choice]

A. Almost no impact

B. Mild impact

C. Moderate impact

D. Serious impact
