# Supplementary material for: The Impact of COVID-19 Pandemic on the Clinical Practice Patterns in Atrial Fibrillation: A Multicenter Clinician Survey in China
Source: J Clin Med. 2022 Oct 31;11(21):6469. doi: 10.3390/jcm11216469 (PMC9653826; doi:10.3390/jcm11216469)
Supplement: Supplementary file 1 [file jcm-11-06469-s001.zip › jcm-1908443-supplementary/Table S1.pdf]

Table S1. Locations of participated cardiologists (n= 213)

Provinces	n (%)
Anhui	9 (4.2)
Beijing	17 (8.0)
Chongqing	5 (2.3)
Fujian	10 (4.7)
Gansu	8 (3.8)
Guangdong	10 (4.7)
Guangxi	5 (2.3)
Guizhou	4 (1.9)
Hainan	8 (3.8)
Hebei	6 (2.8)
Henan	12 (5.6)
Hunan	6 (2.8)
Jiangsu	15 (7.0)
Jilin	5 (2.3)
Liaoning	7 (3.3)
Shangdong	14 (6.6)
Shanghai	27 (12.7)
Shanxi	8 (3.8)
Sichuan	7 (3.3)
Xinjiang	11 (5.2)
Yunnan	9 (4.2)
Zhejiang	10 (4.7)
Total	213 (100.0)
